# Intraparenchymal cerebellar capillary hemangioma in a 32-year-old man: a case report

**DOI:** 10.3389/fsurg.2023.1141522

**Published:** 2023-05-09

**Authors:** Dewa Putu Wisnu Wardhana, Steven Awyono, Christopher Lauren, Rohadi Muhammad Rosyidi, Herman Saputra

**Affiliations:** ^1^ Neurosurgery Division, Department of Surgery, Faculty of Medicine, Academic Hospital of Universitas Udayana, Universitas Udayana, Badung, Indonesia; ^2^ Neurosurgery Division, Department of Surgery, Faculty of Medicine, Prof. Dr. I.G.N.G. Ngoerah General Hospital, Universitas Udayana, Denpasar, Indonesia; ^3^ Department of Neurosurgery, West Nusa Tenggara General Hospital, Medical Faculty of Mataram University, Mataram, Indonesia; ^4^ Department of Anatomical Pathology, Faculty of Medicine, Prof. Dr. I.G.N.G. Ngoerah General Hospital, Universitas Udayana, Denpasar, Indonesia

**Keywords:** capillary hemangioma, immunohistochemistry, intracranial tumor, neurosurgery, oncology

## Abstract

The authors present an unusual case of a 32-year-old adult male with a capillary hemangioma, which developed within the left cerebellar parenchyma. The histopathological examination reveals a mass mostly formed by the proliferation of capillaries, lined by a layer of flat-plump endothelial cells, some branching and dilating large capillaries, forming a lobulated structure separated by fibrocollagenous connective tissue. Immunohistochemistry examination with CD31 and S100 was positive on the endothelial and stromal cells, respectively, and negative S100 on the endothelial cells. Although rare, capillary hemangioma should be one of the differential diagnoses for diagnosing intra-axial lesions in the cerebellar region. Confirmation of the histopathological characteristic is necessary to determine the diagnosis of capillary hemangioma and exclude other differential diagnoses.

## 1. Introduction

Capillary hemangioma is a benign vascular mass or vascular tumor due to abnormal growth of small blood vessels, often found in the skin and connective tissue of neonates or infants and rarely developed in adults. It is reported to have a 1.1%–2.6% prevalence in neonates, especially in the face, scalp, chest, or back area (1, 2). Additionally, intracranial involvement of capillary hemangiomas has been reported rarely, and its exact prevalence is not known. Most of these cases originate from the dura mater and are classified as extra-axial masses. Intra-axial hemangiomas are found less frequently than extra-axial masses (3, 4). To the best of the author's knowledge, no capillary hemangioma has been reported intra-axially within the cerebellar parenchyma.

We present a case report of an intraparenchymal cerebellar capillary hemangioma developed in a 32-year-old man. We provide a detailed description of the clinical history, examination, operative procedure, histopathological findings, postoperative management, and discussion of this case based on previously reported studies and literature.

## 2. Case description

### 2.1. History

A 32-year-old man was brought to the emergency room complaining weakness of all 4 extremities since 1 day prior. This symptoms accompanied by balance disorders. He felt like swaying and difficulty to get up from the bed. He also complained of chronic headaches since five months prior, especially on the back of the head, with characteristics of being tied to a tightrope, intermittent, and only slightly improved with over-the-counter analgesics. There is no history of sensory abnormalities, seizures, nausea, and vomiting. There was no history of weight loss or similar complaints in this patients. There is no history of heredity in the patient's family who suffers from similar complaints or suffers from central nervous system tumors and tumors in other body locations.

The patient underwent a magnetic resonance imaging (MRI) examination at that time. A tumor was found in the left cerebellar area with suspicion of brain tumor with acute hydrocephalus. The patient was advised to undergo a shunt procedure; his symptom improved on the first month after the surgery, there was an improvement in headache and reduced weakness in all four extremities, but a slight balance disturbance was felt.

### 2.2. Examination

We found a normal vital sign with a visual analog scale of 6 and the Karnofsky Performance Scale of 70. A general physical examination revealed no abnormalities. No tumor or vascular lesions were found on the patient's skin such as spider angiomas or other lesions. On neurological examination, it was found that there was a motor weakness in each of the upper and lower extremities, with each MMT score of 3. The patient's physiological reflexes were normal, without any pathological reflex was noted. From sensory examination, no abnormalities were found. On examination of cerebellum functions such as dysdiadochokinesis, heel-knee tests, and forefinger tests, no significant abnormalities were found on these examinations.

**Figure 1:**
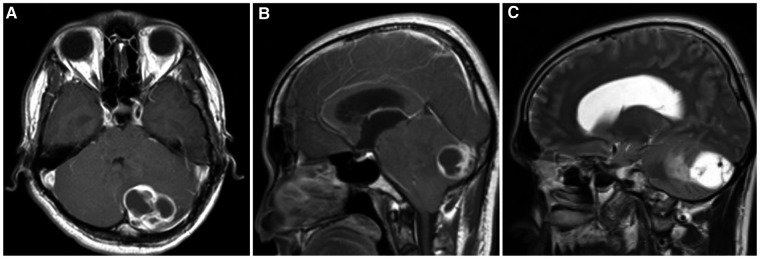
MRI shows a focal mass located in the posterior left cerebellum parenchyma. (**A, B**) Contrast-enhanced (**A**) axial and (**B**) sagittal T1-weighted images. (**C**) T2-weighted image showed similar intensity between the mass and cerebrospinal fluid.

MRI examination revealed intra-axial masses on the left cerebellar parenchyma with a well-defined and contrast-enhanced border (Figures 1A–C). On the T2 sequence, we found multiple septae inside the lesion with a hyperintense feature, which has the same intensity as the cerebrospinal fluids. In addition, we also found dilated ventricle with periventricular edema suggested as hydrocephalus. We then performed a shunt procedure followed by tumor removal.

### 2.3. Operative procedure

**Figure 2:**
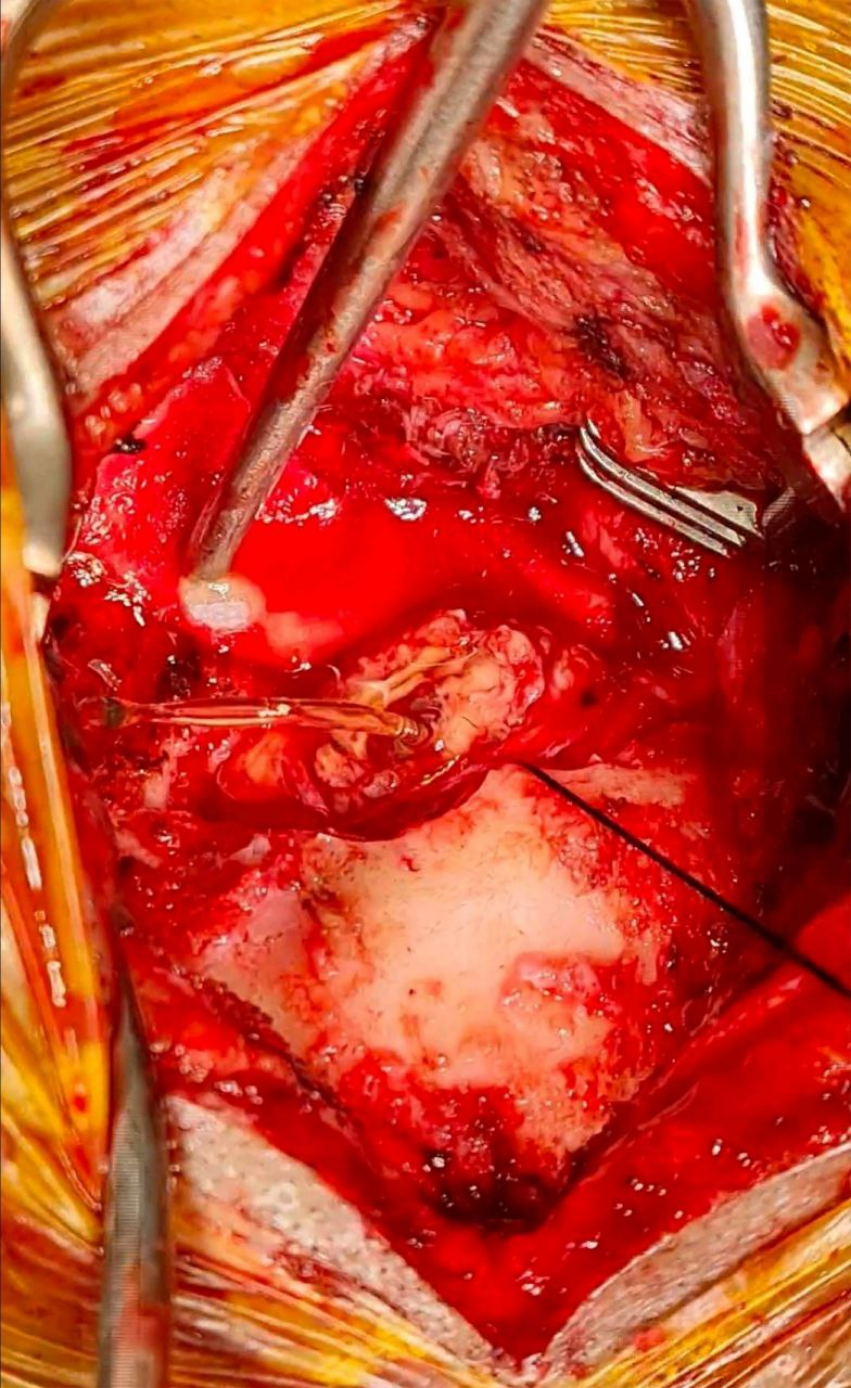
Intraoperative view. The cystic contains bursting out through the corticotomy side due to high pressure.

We performed a midline suboccipital craniotomy to expose the tumor. Dura was incised using Y-shaped incision and exposed severely edema of the cerebellum. tumor removal begin from the puncate over the left cerebellar cortex. A reddish wall cystic mass appeared with a highly vascular configuration on the posterior surface of the left cerebellum. A yellowish liquid appears on the inside of the mass and due to high intracranial pressure the cystic contain burst out through the corticotomy side (Figure 2). The mass was found adjacent to the left transverse sinus. Tumor removal was performed in such manner to preserve the transverse sinus. After performing gross total removal of the tumor, adequate hemostasis was achieved using bipolar and hemotstatic agent. The dura was then closed using a watertight fashion. The nuchal muscles and ligaments were reconstruct to achieved good craniovertebral muscle stabilization, followed by closing of the skin layer by layer. The mass was sent for further pathological examination.

### 2.4. Histopathological findings

**Figure 3:**
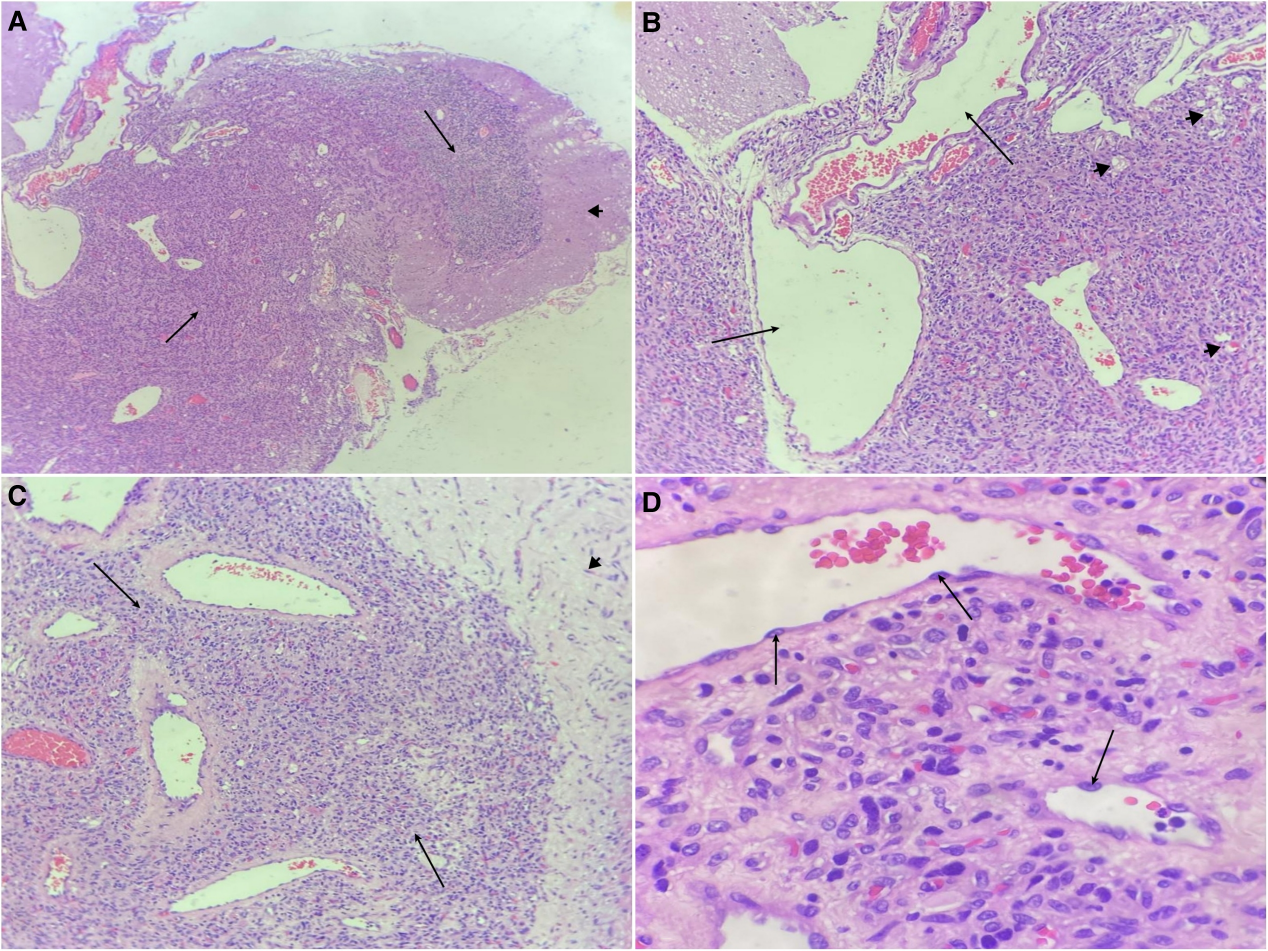
Microscopic view. (**A**) The tumor mass (black arrow), surrounded by cerebellar tissue (arrowhead) (HE stain; 40 × magnifications). (**B**) Branching and dilating large capillaries (black arrow) with small capillaries (arrowhead) (HE stain; 100 × magnifications). (**C**) The blood vessels form a lobulated structure (black arrow), surrounded by cerebellar tissue (arrowhead) (HE stain; 100 × magnifications). (**D**) The blood vessels are lined by a layer of flat-plump endothelial cells (black arrow) (HE stain; 400 × magnifications).

**Figure 4:**
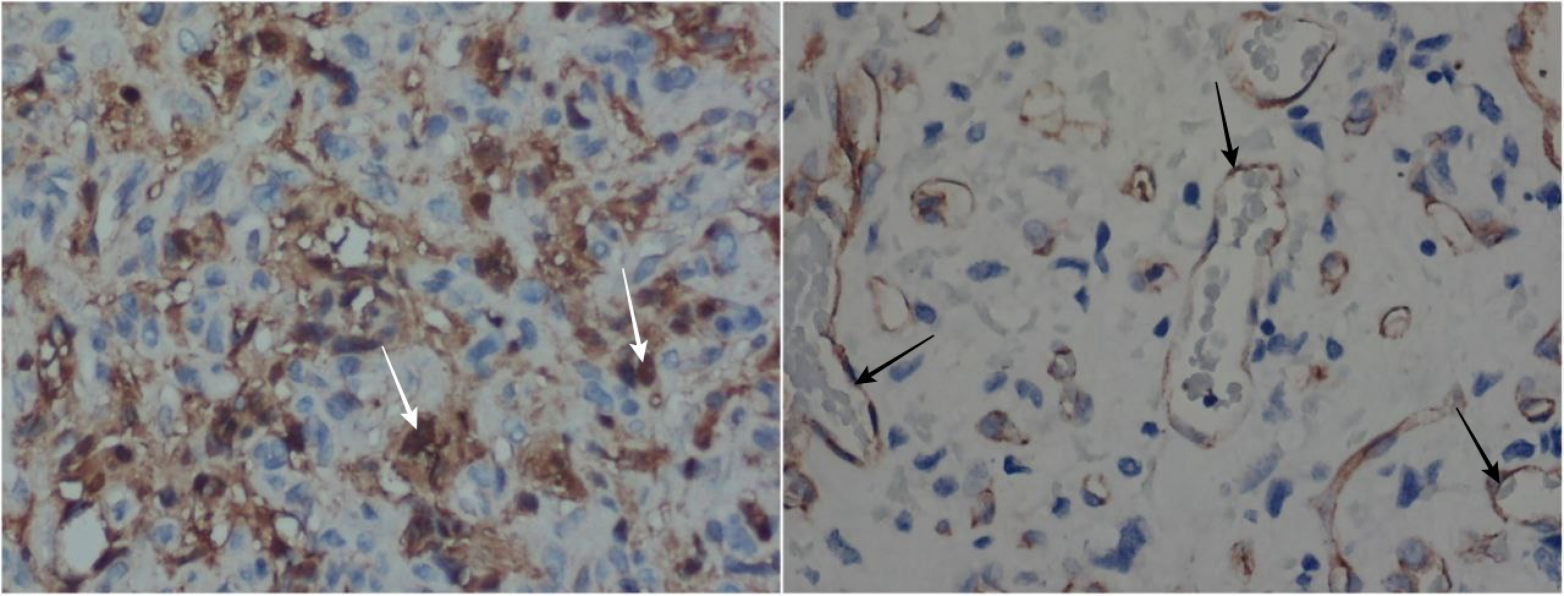
The IHC results with S100 show positive results on the stromal cells (white arrow) but negative on endothelial cells (400 × magnifications) (left). The CD31 shows positive results on the endothelial cells (black arrow) (400 × magnifications) (right).

On the macroscopic cross-section, the mass was grayish-white mixed with red color, measured around 3  ×  2  ×  0.2 cm in diameter with a wall thickness of 0.1–0.2 cm. On microscopic cross-sections, the mass appears to be well-defined with the surrounding tissue without being covered by a capsule (Figure 3A). The mass is formed by the proliferation of capillaries, which are lined by a layer of flat-plump endothelial cells with some of the lumen filled with erythrocytes (Figure 3D. Pericytic cells are seen under the endothelial cells of the blood vessels. These blood vessels form a lobulated structure that varies, where each part is separated by fibro collagenous connective tissue (Figure 3C). There are some large capillaries, following the description of the parent vessels, where branching and dilatation are visible (Figure 3B. Some features of mitosis can also be found. Based on the histopathological description, it can be concluded that the mass shows the characteristics of capillary hemangioma. The specimen was examined under a microscope with hematoxylin-eosin (HE) staining and 40x, 100x, and 400 × magnification. These findings were supported by immunohistochemistry (IHC) examination for S100 and CD31 of the mass. It reveals positive CD31 on the endothelial cells and S100 on the stromal cells with negative S100 on the endothelial cells (Figure 4). These results conclude that the mass was truly a blood vessel tumor, consistent with a hemangioma.

### 2.5. Postoperative management

The patient was hospitalized for seven days, and during postoperative course, the patient felt symptoms improvement especially the increase in muscle power over the all four extremities. The patient could barely walk with assistive devices such as a cane on day fifth day after the surgery. The patient was discharged on day seven without any associated symptoms or new neurological deficits. The patient was only given analgesia as a home remedy, accompanied by routine control two times weekly with routine physiotherapist exercise. One month after surgery he can easily mobilize without any assistive device.

## 3. Discussion

Capillary hemangioma is a benign tumor consisting of abnormal growth of capillaries. Usually, they appear in the first six months of life. It grows rapidly until it reaches 12 months of age and usually undergoes complete spontaneous regression by five years (2). Capillary hemangiomas in infants are most commonly manifested on the skin, with an estimated frequency of 10% in the first year of life (5). This tumor has rarely been reported to arise in the intracranial cavity (6). Koga et al. cataloged 36 cases of intracranial capillary hemangiomas from around the world, which were reported in the literature (Supplementary Material Table S1). Of the 36 reported cases, only 5 cases were found to be intra-axial, and 3 cases were found to be in adult males (4). To the best of the author's knowledge, no capillary hemangioma has been reported intra-axially within the cerebellar parenchyma.

Demographically, capillary hemangioma is more often found in women and occurs in young adult age (7). These tumours more often manifest as benign tumours around the periorbital area, rather than as lesions in the intracranial area (8). Other intracranial lesions such as hemangioblastoma, where this tumour is the primary tumor in the cerebellum area, have different demographic characteristics compared to capillary hemangioma (9–11). In hemangioblastoma, this lesion is more common in older patients, in the age range of 60–79 years, and less frequently in young adults. In contrast to capillary hemangioma, CNS hemangioblastoma is commonly found in males (10).

It is not known whether there is a direct or indirect genetic relationship with the occurrence of capillary hemangioma in the intracranial area, because this case is still very rare. However, it has been found that genetic factors are involved in capillary hemangioma in extracranial locations. Some of these genes, such as mutation of p.Glu70Lys and p.Trp88Ter, have a risk of causing a capillary hemangioma (12). In CNS hemangioblastoma, the disease is often related or associated with Von Hipple-Lindau disease, so it is related to mutations in the VHL gene, namely Exons 1, 2, and 3 (11, 13). In addition, hemangioblastoma can also be accompanied by the involvement of other organ lesions. Other vascular lesions, such as cavernoma, can be caused by mutations in the CCM1/KRIT1, CCM2/MGC4607, or CCM3/PDCD10 genes (14, 15). In AVM the involvement of genetic factors is still unclear (16).

In capillary hemangioma, patients most often complain of headache (40%) as the main symptom. This complaint was followed by cranial nerve palsies (30%), visual disturbances (19%), nausea, vomiting (17%), seizures (13%), hydrocephalus (13%), limb motor weakness (13%), to decreased consciousness (6%) (7). In our case, this patient experienced progressive headaches, and motor weakness in all extremities, with the presence of hydrocephalus. Hemangioblastoma is usually associated with impaired cerebellum function and signs of increased ICP, such as gait ataxia (64%), dysmetria (64%), headache (12%), diplopia (8%), vertigo (8%) to vomiting (8%) (11). Other vascular lesions such as AVM and cavernoma can cause clinical manifestations, especially if the blood vessels involved are ruptured, ranging from bleeding and spasms, followed by headaches, and neurological deficits, to nausea and vomiting (14, 17).

Capillary hemangioma is often difficult to diagnose if only relying on radiological examination because of its rarity, and the pathognomonic picture is not so clear. However, based on the literature, this lesion can be suspected based on the typical radiological appearance (6, 18, 19). Imaging features seen in cases of capillary hemangioma is an enhanced mass, giving a characteristic of high vascularity. Usually, this lesion shows a homogeneous contrast enhancement. In contrast to the cases we encountered, on the T1 image, there is a cystic mass lesion with heterogeneous contrast enhancement and a hyperintense wall with a hypointense interior of the lesion, which can be associated with intra-tumoral hemorrhage or necrosis (20). On the T2 image, multiple hypointense images inside the lesion indicate the presence of flow voids.

Determination of the diagnosis of capillary hemangioma is usually seen based on histopathological features and immunohistochemical examination. The histopathological features usually found in capillary hemangiomas are a dense proliferation of numerous small blood vessels with endothelial cells, lobular in shape, and some intratumoral hemorrhage (4, 21). In our case, we found a mass that consisted of a proliferation of capillaries, which form a lobulated structure, separated by fibrocollagenous connective tissue, and a partial picture of the parent vessels in the form of large capillaries. An IHC examination can be done by examining a cluster of differentiation (CD) 31 and CD34, which can clearly show the picture of the vascular architecture (6). In our case, IHC for CD31 and S100 was performed. CD31 and S100 were positive on the endothelial and stromal cells, respectively, and negative S100 on the endothelial cells. These results conclude that this specimen was consistent with hemangioma, a blood vessel tumor.

Several cases can be used as a differential diagnosis of this case, especially the other lesions involving the cerebellar parenchyma. Lesions that often arise in the cerebellum area, especially those affecting adult men, can be hemangioblastomas, gliomas, or metastatic processes from other locations. The three differential diagnoses are the main causes of cerebellar intra-axial tumor and have several radiological features similar to the patient in our case. One way to diagnose and provide appropriate therapy is to take the tumor tissue and examine the histopathological feature. In hemangioblastoma, there is a well-defined cystic mass with enhancing mural nodules, accompanied by neoplastic stromal cells with foamy cytoplasm and a structure of many branching small blood vessels (22). We suspected this mainly because of the absence of homogeneous enhancement with intravenous contrast administration and cystic appearance of the mass on MRI examination. Glioma is rare in adults and more common in young children. However, the radiological picture gives a characteristic picture that can indicate the possibility of glioma as the main cause of the lesion (23). The metastatic process is also one of the main differential diagnoses for masses involving the cerebellar area. Although this patient does not have any history of a primary tumor in another location due to the high incidence of metastasized lesions in the cerebellar region, we should consider this lesion's possibility. The possibility of metastases process is one of the main reasons for resection and further treatment of this patient (6, 24, 25).

In general, patients with capillary hemangioma, whether total or partial resection, have a good outcome. Most of the patients marked excellent improvement in neurological status. This was described in a study conducted by Santoro et al., where patients who underwent total resection, found an improvement in neurological status in 66% of cases and partial resection found improvement in 55% of cases (7). In tumours that were completely resected, no recurrence was found in all cases.

### 3.1. Patient perspective

In our case, the patient initially did not seek for doctor's treatment. He felt a mild headache and did not pay much attention to it. However, as the tumor grow and lead to high intracranial pressure, the headache gets worsened with general weakness which prompted the patient to seek further medical attention and then planned for cerebral MRI. It revealed that he had a tumor on the cerebellum which compressed the CSF flow and led to hydrocephalus. The patient was advised to undergo surgery for tumor removal. Initially, the patient refused and asked to negotiate first, but after deliberating for 2 days the patient agreed with the action to be taken. After surgery, the patient was treated in the intensive care unit for 1 day and because his condition was stable, he was then transferred to the surgical ward. The motor condition and complaints of headache gradually got better, until the 5th postoperative day the patient was able to walk with the help of a cane. The patient was greatly helped by the operative action and appreciated the surgical team. The patient was allowed to go home on the 7th postoperative day without finding any weakness in the extremities. Likewise, during follow-up within the first 1 month, the patient felt that his complaints were gradually improving and he was able to walk again without the help of a cane. This condition causes the patient to feel grateful for the choice of therapy that has been given because the clinical condition is also getting better compared to the initial conditions when admitted to the hospital.

## 4. Conclusion

Intraparenchymal cerebellar capillary hemangioma is an unusual finding. To the best of the author's knowledge, no capillary hemangioma has been found in the cerebellar parenchyma as in our case, neither in journals nor in other scientific literature. Although rare, lobular capillary hemangioma should be one of the differential diagnoses for diagnosing intra-axial lesions in the cerebellar region. Resection, followed by histopathological and IHC examination, is the necessary management in determining the diagnosis of capillary hemangioma.

## Data Availability

The original contributions presented in the study are included in the article/**Supplementary Material**, further inquiries can be directed to the corresponding author.
