# Supplementary material for: Intraparenchymal cerebellar capillary hemangioma in a 32-year-old man: a case report
Source: Front Surg. 2023 May 9;10:1141522. doi: 10.3389/fsurg.2023.1141522 (PMC10203496; doi:10.3389/fsurg.2023.1141522)
Supplement: Supplementary file 1 [file Table1.docx]

Supplementary Material

Intraparenchymal Cerebellar Capillary Hemangioma in a 32-Year-Old Man: a Case Report

Dewa Putu Wisnu Wardhana ^1*^, Steven Awyono ^2^, Christopher Lauren ^2^, Rohadi Muhammad Rosyidi ^3^, Herman Saputra ^4^

*** Correspondence:** Dewa Putu Wisnu Wardhana: wisnu_wardhana@unud.ac.id

# Supplementary Tables

**Table 1.** Reported cases of intracranial capillary hemangioma (Abbreviations: M: Male; F: Female)

|  | **Authors** | **Year** | **Age** | **Sex** | **Origin** | **Treatment** | **Pathology** |
| --- | --- | --- | --- | --- | --- | --- | --- |
| **Extra-axial mass** | | | | | | | |
| 1 | Willing et al | 1993 | 1 year | M | Convexity | Resection | Yes |
| 2 | Watanabe et al | 2001 | 8 years | M | Middle cranial fossa | Resection | Yes |
| 3 | Tsao et al | 2003 | 15 years | F | Middle cranial fossa | Radiosurgery |  |
| 4 | Tsao et al | 2003 | 19 years | F | Middle cranial fossa | Radiosurgery |  |
| 5 | Abe et al | 2004 | 8 years | M | Middle cranial fossa | Resection | Yes |
| 6 | Simon et al | 2005 | 31 years | F | Cerebellar tentorium | Resection | Yes |
| 7 | Le Bihannic et al | 2005 | 1.5 months | M | Anterior choroidal artery | None | Yes |
| 8 | Brotchi et al | 2005 | 10 years | F | Convexity | Resection | Yes |
| 9 | Karikari et al | 2006 | 3 months | M | Fourth ventricle | Resection | Yes |
| 10 | Smith et al | 2007 | 26 years | F | Middle cranial fossa | Resection | Yes |
| 11 | Uyama et al | 2008 | 4 months | F | Convexity | Resection | Yes |
| 12 | Daenekindt et al | 2008 | 2 months | M | Middle cranial fossa | Resection | Yes |
| 13 | Maure et al | 2010 | 44 years | F | Convexity and middle cranial fossa | Resection | Yes |
| 14 | Lee et al | 2010 | 59 years | F | Infundibular recess | Biopsy | Yes |
| 15 | Phi et al | 2012 | 8 years | M | Convexity | Resection | Yes |
| 16 | Phi et al | 2012 | 13 years | M | Cerebellar tentorium | Resection | Yes |
| 17 | Phi et al | 2012 | 30 years | F | Cerebellar tentorium | Resection | Yes |
| 18 | Phi et al | 2012 | 44 years | F | Ethmoid and sphenoid sinuses | Resection | Yes |
| 19 | Morace et al | 2012 | 26 years | F | Cavernous sinus | Resection/radiation | Yes |
| 20 | Morace et al | 2012 | 61 years | F | Cavernous sinus | Resection/radiation | Yes |
| 21 | Morace et al | 2012 | 14 years | M | Middle cranial fossa | Resection/radiation | Yes |
| 22 | Morace et al | 2012 | 42 years | M | Convexity | Resection | Yes |
| 23 | Zheng et al | 2012 | 3 years | M | Middle cranial fossa | Resection | Yes |
| 24 | Mirza et al | 2013 | 28 years | F | Cerebellar tentorium | Resection | Yes |
| 25 | Mirza et al | 2013 | 41 years | F | Convexity | Resection | Yes |
| 26 | Jalloh et al | 2014 | 0.5 months | M | Middle cranial fossa | Resection | Yes |
| 27 | Okamoto et al | 2015 | 82 years | F | Convexity | Resection | Yes |
| 28 | Nepute et al | 2016 | 40 years | M | Petrous bone | Resection | Yes |
| 29 | Xia et al | 2017 | 33 years | F | Cerebellar tentorium | Resection | Yes |
| 30 | Low et al | 2017 | 64 years | F | Cavernous sinus | Biopsy | Yes |
| 31 | Almaghrabi et al | 2018 | 59 years | F | Convexity | Resection | Yes |
| **Intra-axial mass** | | | | | | | |
| 1 | Abe et al | 2004 | 20 years | M | Subcortical | Resection | Yes |
| 2 | Abe et al | 2004 | 16 years | F | Subcortical | Resection | Yes |
| 3 | Younas et al | 2011 | 69 years | M | Subcortical and basal ganglia | Resection | Yes |
| 4 | John et al | 2012 | 59 years | M | Subcortical | Resection | Yes |
| 5 | Koga et al | 2019 | 15 years | F | Subcortical | Resection | Yes |
| 6 | Present case | 2022 | 32 years | M | Subcortical | Resection | Yes |
